# The genetic diversity of *Borrelia afzelii* is not maintained by the diversity of the rodent hosts

**DOI:** 10.1186/s13071-018-3006-2

**Published:** 2018-08-06

**Authors:** Claudia E. Coipan, Gilian L. A. van Duijvendijk, Tim R. Hofmeester, Katsuhisa Takumi, Hein Sprong

**Affiliations:** 1 Laboratory of Entomology, Wageningen University, Wageningen, The Netherlands; 2 Centre for Epidemiology and Surveillance of Infectious Diseases, National Institute for Public Health and Environment (RIVM), Bilthoven, The Netherlands; 3 Applied Biology, HAS University of Applied Sciences, ‘s Hertogenbosch, The Netherlands; 4 Resource Ecology Group, Wageningen University, Wageningen, The Netherlands; 5 Department of Wildlife, Fish and Environmental Studies, Swedish University of Agricultural Sciences, Umeå, Sweden; 6 Laboratory for Zoonoses and Environmental Microbiology, National Institute for Public Health and Environment (RIVM), Bilthoven, The Netherlands

**Keywords:** *Borrelia burgdorferi* (*s.l.*), *Ixodes ricinus* larvae, Rodents, IGS, *ospC*, *dbpA*

## Abstract

**Background:**

Small mammals are essential in the enzootic cycle of many tick-borne pathogens (TBP). To understand their contribution to the genetic diversity of *Borrelia afzelii*, the most prevalent TBP in questing *Ixodes ricinus*, we compared the genetic variants of *B. afzelii* at three distinct genetic loci. We chose two plasmid loci, *dbpA* and *ospC*, and a chromosomal one, IGS.

**Results:**

While the larvae that fed on shrews (*Sorex* sp.) tested negative for *B. afzelii*, those fed on bank voles (*Myodes glareolus*) and wood mice (*Apodemus sylvaticus*) showed high infection prevalences of 0.13 and 0.27, respectively. Despite the high genetic diversity within *B. afzelii*, there was no difference between wood mice and bank voles in the number and types of *B. afzelii* haplotypes they transmit.

**Conclusions:**

The genetic diversity in *B. afzelii* cannot be explained by separate enzootic cycles in wood mice and bank voles.

**Electronic supplementary material:**

The online version of this article (10.1186/s13071-018-3006-2) contains supplementary material, which is available to authorized users.

## Background

*Borrelia burgdorferi* (*sensu lato*) is a bacterial complex whose members are causative agents of Lyme borreliosis, the most widespread vector-borne disease in Europe. The genospecies that accounts for the majority of Lyme borreliosis cases is *Borrelia afzelii* [1]. The enzootic transmission cycle of *B. afzelii* predominantly involves small mammals and *I. ricinus* ticks, in which it is also the most prevalent *Borrelia* genospecies [2, 3].

The high genetic diversity of *B. burgdorferi* (*s.l.*), at both inter- and intra-specific level, is partly the reason for the multiple clinical manifestations that the infection with these bacteria can display in humans [4–7]. An important role in the maintenance of the intraspecific genetic diversity of *B. burgdorferi* (*s.l.*) has been attributed to the host community. One of the underlying mechanisms is the multiple-niche polymorphism balancing selection where various vertebrate hosts can act as ecological niches for some genotypes of a species [8, 9]. This balancing selection entails that the bacteria have different fitness in different vertebrate hosts. Changes in the vertebrate host composition could therefore result in changes in the abundance of the more pathogenic bacteria [10–12].

Genes such as the outer surface protein C gene (*ospC*) and ribosomal protein L2 gene (*rplB*) have been used in studies addressing the genetic diversity of *B. afzelii* [13–16] in either skin biopsies from rodents, or questing *I. ricinus*. While rodents could get infected by certain *Borrelia* genotypes, it is not necessary that they transmit them with equal efficiencies to the ticks feeding on them. Likewise, the presence of the genotypes with multiple sources in questing ticks cannot indicate which of the sources contributed most to the abundance of those respective genotypes. A manner to overcome these issues is to study the distribution of the various genotypes in the engorged larvae attached to their hosts. This has been done in two European studies highlighting the existence of co-infection with multiple *ospC* haplotypes of *B. afzelii* in fed larvae [17, 18]; these studies, however, did not address the question of differential roles of small mammal species in the maintenance of *B. afzelii* genetic polymorphism. To our knowledge, the only comparative studies on fed larvae from various rodent species were performed on *B. burgdorferi* (*sensu stricto*) (*s.s.*) [19, 20]. There they found that certain genotypes of *B. burgdorferi* (*s.s.*) are associated to certain vertebrate hosts, supporting the hypotheses that host adaptation is an important determinant for the genetic differentiation of *B. burgdorferi* (*s.s.*) and potentially also for its variation in pathogenicity in humans.

In this study, we tested the hypothesis of host driven multiple-niche polymorphism of *B. afzelii*. We did this by testing fed larvae collected from five small mammal species: the wood mouse (*Apodemus sylvaticus*), bank vole (*Myodes glareolus*), field vole (*Microtus agrestis*), common shrew (*Sorex araneus*) and pygmy shrew (*Sorex minutus*). While the first two are among the most abundant rodent species in northwestern Europe [21–23] and recognized as the most important reservoirs for *B. afzelii* [3, 24], the field vole and shrews are less studied in this context. To this end, we chose two protein-coding genes, whose products are active elicitors of the immune response of the rodent hosts [25, 26]: *ospC* and decorin binding protein A (*dbpA*). According to the niche-polymorphism model, we expected to find distinct haplotypes of the genes in the larvae collected from the small mammals. In order to be able to identify the *Borrelia burgdorferi* (*s.l.*) genospecies we additionally used the 5S-23S intergenic spacer region (IGS), which is located on the chromosome and is evolving neutrally [27].

## Methods

### Sample collection

We trapped rodents and shrews with small mammal live-traps (Heslinga Traps, Groningen, The Netherlands) baited with grain, carrot and mealworms (as in [28]). Traps were checked at 12 h intervals. The sampling was performed between July 2013 and August 2014 at 11 different sites located in ten forests in The Netherlands (Additional file 1: Table S1). Each animal was thoroughly examined for ticks and then released at the site of capture. Animals that accidentally died in the traps were brought to the laboratory for a thorough examination for ticks (see Additional file 2: Table S2 for an overview of which animals were checked alive, and which were checked dead). Each tick was collected and placed in a separate tube. For each tick we registered the species, the development stage (larva, nymph, adult) and the feeding status (fed/unfed). An overview of the tick burdens per species can be found in Additional file 3: Table S3. The samples were stored at -20 °C until DNA extraction.

### DNA extraction and qPCR detection of microorganisms

Total DNA was extracted from engorged larvae only. The level of engorgement was determined based on visual inspection under a stereomicroscope, where the flat larvae were assigned as unfed, while the partly- or fully-fed were assigned as engorged. This was done to ensure that the *Borrelia burgdorferi* (*s.l.*) detected in the larvae came from the vertebrate hosts they were feeding on at that moment, considering that transovarial transmission of these bacteria is absent or very low [29, 30]. The extraction was performed using the Qiagen DNeasy 96 Blood & Tissue Kit (Qiagen, Venlo, The Netherlands).

For the detection of *B. burgdorferi* (*s.l.*), a triplex qPCR, targeting *ospA* and *flaB* genes of *B. burgdorferi* (*s.l.*) and the *flaB* gene of *B. miyamotoi*, was used. The sequences of primers and probes are given in Additional file 4: Table S4. qPCRs were performed using the iQ Multiplex Powermix PCR reagent kit (Bio-Rad Laboratories, Hercules, USA), in a LightCycler 480 Real-Time PCR System (F. Hoffmann-La Roche, Basel, Switzerland). The triplex reaction mix for *Borrelia* species consisted of iQ multiplex Powermix, 100 nM of the B-FlaB-Rc and B-FlaB-Rt primers, 200 nM of the B-FlaB-F, FlabBm.motoiF2, and FlabB.m.motoiR3 primers, 400 nM of the B-OspA_modF and B-OspA_borAS primers, 100 nM of the B-OspAmodPatto probe, 200 nM of the B-FlaB-Patto and FlabBm.motoiPro probes, and 3 μl of template DNA in a final volume of 20 μl. Analysis was performed using the second derivative calculations for crossing point values. For each run three positive controls, two negative controls, and two blank samples were included.

### Conventional PCR and sequencing

We targeted three genes located on different genetic elements of *B. afzelii*: (i) 5S-23S intergenic spacer region (IGS) located on the chromosome, (ii) *dbpA* located on plasmid lp54, and (iii) *ospC* located on plasmid cp26. In order to build a phylogeny of the spirochetes found in the rodent ticks, we used the 5*S-*23*S* rDNA intergenic spacer (IGS). The PCR was performed according to the protocol described in Coipan et al. [27].

Conventional PCR for *ospC* was performed using the forward primer (OC6) described by Qiu et al. [31] and a modification of the reverse primer (OC602) described by the same author, in a final concentration per reaction of 0.4 μM. The sequences of the primers are given in Additional file 4: Table S4. The PCR reaction was done using the HotStar Master Mix Kit (Qiagen), with the following conditions: 15 min at 94 °C, then cycles of 20 s at 94 °C, 30 s at 70 °C, 30 s at 72 °C lowering the annealing temperature 1 °C each cycle until reaching 60 °C, then 40 cycles at this annealing temperature and ending with 7 min at 72 °C. Due to the high sequence variability of *dbpA*, the conventional PCR for this gene was performed using three primer pairs, described in Additional file 5: Table S5. The PCR reaction mix and conditions were the same as for *ospC*.

PCR products were sequenced using an ABI PRISM BigDye Terminator Cycle sequencing Ready Reaction kit (Applied Biosystems, Foster City, California). Sequences were confirmed by sequencing both strands [32]. Storage, trimming and cleaning of the sequences were performed in BioNumerics version 7.0 (Applied Math, Sint-Martens-Latem, Belgium).

### Genetic analysis

Sequences were aligned by MAFFT v.7.222 [33]. Phylogenetic trees of all three genes were constructed by using a maximum likelihood algorithm, in RAxML, run under RDP 4.56 [34], with 100 bootstraps. Based on the clustering in the phylogenetic trees each allele of the three genes received a unique ID number/name.

The *ospC* alleles were named according to Durand et al. [15]. To identify similarities with sequences from other areas of Europe we downloaded IGS, *dbpA* and *ospC* sequences available in GenBank. The phylogenetic trees of IGS and *ospC* were rooted with *B. garinii*. For data visualization we constructed phylogenetic trees linked with heatmaps of the rodent species, using the functions implemented in the *ggtree* package [35].

We tested for genetic differentiation between the various sites and rodent species using the AMOVA test implemented in Arlequin 3.5 [36], where we defined the rodent species as a group and the various sampling locations as populations. We used R 3.4.4 to test for differences in tick burden using a generalized linear model with a negative binomial distribution and a log-link function [37].

## Results

Full body inspection for ticks was performed on 120 small mammals (Additional file 2: Table S2). The small mammals carried a total of 4292 larval and 94 nymphal *Ixodes ricinus*, with variable infestation levels between the various species (Additional file 3: Table S3). Wood mice did not have a significantly higher mean larval burden (mean ± SD, 48.1 ± 51.9) than bank voles (32.2 ± 39) (nbGLM: *Z* = 1.8, *P* = 0.08). Field voles had the highest mean larval burden (91.2 ± 165.5), which was higher than bank voles (nbGLM with bank voles: *Z* = 2.06, *P* = 0.05) but not than wood mice (nbGLM with wood mice; *Z* = 1.24, *P* = 0.22).

We tested for the presence of *Borrelia burgdorferi* (*s.l.*) in 1431 fully engorged larvae from 114 small mammals (Additional file 2: Table S2). On six of the common shrews and one of the wood mice we found only unfed larvae. Larvae from three of the five investigated small mammal species were positive, with an overall infection prevalence of 17.8% (255/1431). The positive larvae came from 29.8% (34/114) of the investigated vertebrates (Table 1). All but one *B. burgdorferi* (*s.l.*) typed by sequencing of IGS were identified as *B. afzelii*. The exception was identified as *B. garinii* in one bank vole. The prevalence of infection with *B. afzelii* was significantly higher in larvae fed on bank voles than on wood mice (Fisher’s exact test, *P* < 0.001). This also held true for the two sites from which the majority of our rodents came from: Noord Ginkel (Fisher’s exact test, *P* < 0.001) and Planken Wambuis (Fisher’s exact test, *P* < 0.001). In one site (Deelerwoud) the prevalence of infection was higher in wood mice (Fisher’s exact test, *P* < 0.02). Of the 255 qPCR positive larvae, 163 yielded a sequence for IGS, 165 for *dbpA* and 164 for *ospC* (sequences given in Additional file 6: Table S6).

**Table 1:** Tick burden and prevalence of infection of the five small mammalian species

	Larval/nymphal tick burden median (range)	*B. burgdorferi* (*s.l.*) prevalence of infection (positive/tested)	
		Larva	Rodent
*A. sylvaticus*	24.5/0 (1–226/0–7)	0.13 (98/733)	0.33 (16/48)
*M. glareolus*	16/0 (1–153/0–15)	0.27 (152/570)	0.34 (15/44)
*Mi. agrestis*	30/2 (2–386/1–9)	0.42 (5/12)	0.6 (3/5)
*S. araneus*	2/0 (0–28/0–1)	0 (0/113)	0 (0/15)
*S. minutus*	2.5/0 (2–3/0–0)	0 (0/3)	0 (0/2)

**Fig. 1:**
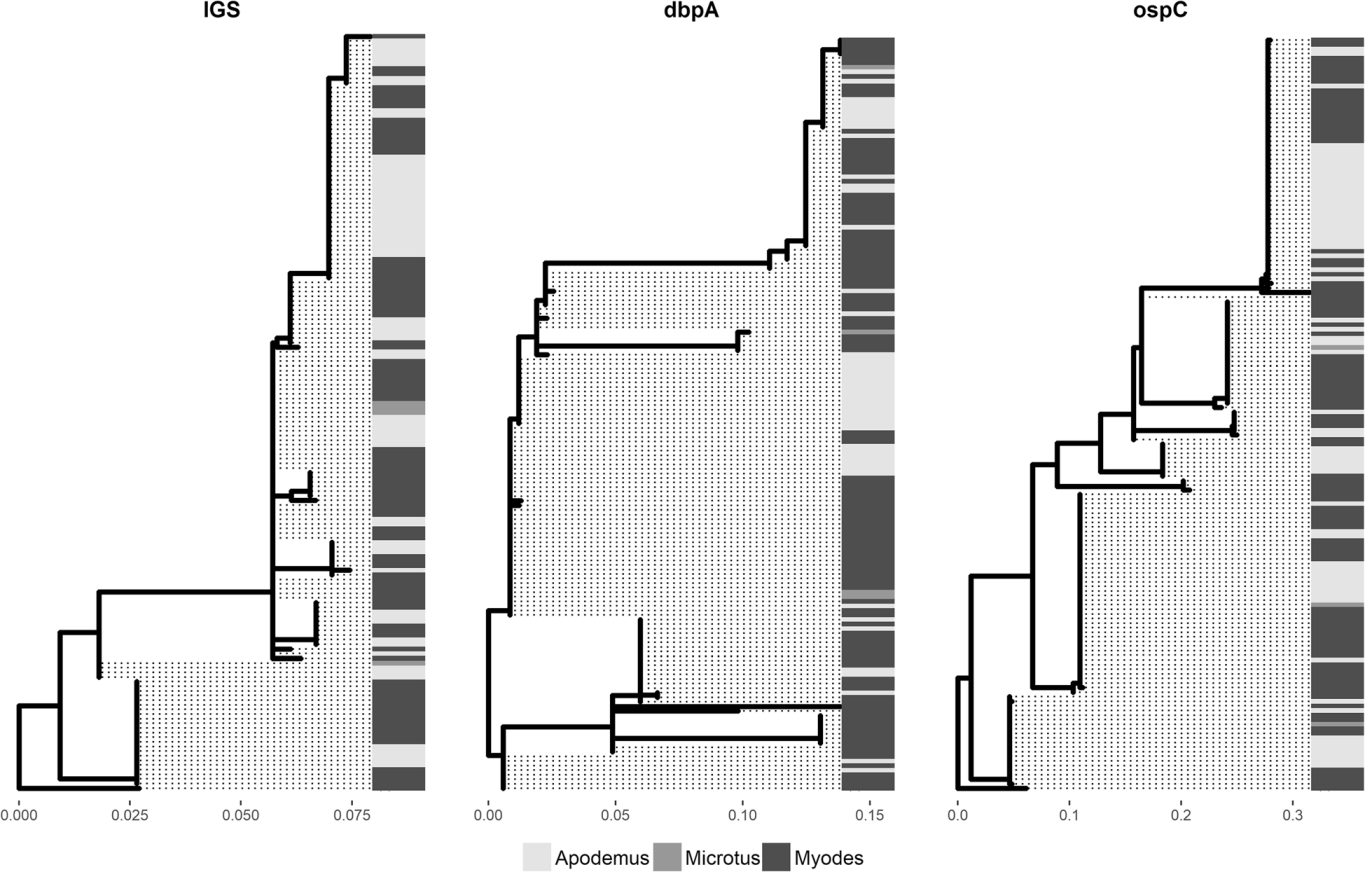
Phylogenetic trees of IGS, *dbpA* and *ospC*. The associated heatmaps depict the rodent genus from which the ticks were collected: *Apodemus sylvaticus*, *Myodes glareolus* and *Microtus agrestis*; *B. garinii* sequences were used for rooting the IGS and *ospC* trees

We identified 11 haplotypes of IGS, 9 haplotypes of *dbpA* and 9 haplotypes of *ospC* (Additional file 7: Table S7). The majority of the haplotypes could be found in both larvae that fed on wood mice and larvae that fed on bank voles (Fig. 1). Most of the haplotypes that were associated with only one of the two rodent species were present at low frequencies, very often being found in one tick only (Additional file 6: Table S6). For *ospC*, seven of our nine haplotypes were described in a recent article by Durand et al. [15]. Similarly, IGS and *dbpA* haplotypes identical to the ones described in this study were identified in other European countries (results not shown). The sequences in our dataset containing ambiguous nucleotides were inferred to be the result of coexistence of the already defined haplotypes, and not new ones, since the double peaks occurred at the same positions as the segregating sites in the unambiguous sequences.

Most of the rodents allowed transmission of multiple *Borrelia* haplotypes, with no difference between the bank voles and the wood mice in the mean number of haplotypes they can transmit (t-test: IGS, *t*_(25.7)_ = -1.68, *P* = 0.11; *dbpA*, *t*_(16.3)_ = -1.12, *P* = 0.9; *ospC*, *t*_(21.6)_ = -1.29, *P* = 0.21). The AMOVA test indicated that there was no genetic differentiation between the *B. afzelii* genotypes carried by the two rodent species (Table 2) for any of the three investigated loci, the percentage of variation explained by the rodent species being less than 10%. The negative variance components observed for *dbpA* and *ospC* indicate the absence of genetic structure and should be interpreted as zero. The largest percentage of the variation was found within the population of *B. afzelii* at each location. There was also a relatively high variation among the various sampling locations (29.69–44.77).

**Table 2:** Results of AMOVA test for the three selected genes

Gene	Percentage of variation (degrees of freedom)		
	Between genera	Between samples within genus	Within samples
IGS	5.32 (2)	29.69 (10)	65 (139)
*dbpA*	-11.78 (2)	39.69 (8)	72.09 (135)
*ospC*	-0.69 (2)	44.77 (11)	55.92 (141)

## Discussion

In this study, we tested whether the three most common rodent species in northwestern Europe transmit different variants of *B. afzelii*, by comparing the haplotypes of *B. afzelii* at three distinct genetic loci in engorging larvae removed from rodents. Two main assumptions were made in the analysis of the data: (i) the newly hatched larvae that are questing for a vertebrate host are *B. burgdorferi* (*s.l.*) free, as previous studies have shown transovarial transmission to be close to null [38, 39] and (ii) all larvae have been attached to their hosts long enough to get infected with the bacteria of interest for our study [38]. Recent studies [29] have shown that transovarial transmission of *B. burgdorferi* (*s.l.*) might be the reason that 0.62% of the larvae are infected. We argue that, at this prevalence, the transovarially transmitted *Borrelia burgdorferi* (*s.l.*) in our study would represent only a minor fraction (8–9 positive larvae) and would, therefore, not be a major bias in our results.

Our comparison of the infection prevalence in the larvae feeding on the various small mammals indicated that *B. afzelii* is more prevalent in larvae feeding on bank voles than in those feeding on wood mice. While previous studies have indicated this with xenodiagnostic data [40, 41], our data confirm that the bank voles are better transmitters of *B. afzelii* than wood mice using field data.

We also find that bank voles and wood mice transmit the same *B. burgdorferi* (*s.l.*) genotypes to the larvae they feed. The existence of multiple genotypes with divergent nucleotide sequences at several loci in the genome of *Borrelia* could be explained, among others, by the multiple-niche polymorphism balancing selection, where the bacteria have evolved to be adapted to various vertebrate hosts. *Borrelia burgdorferi* (*s.s.*) bacteria in North America have been shown to confirm to this hypothesis, with host-specific bacterial strains [19, 20]. The same hypothesis was tested on the European *B. afzelii* using tissues from various rodent species [13, 14] but the results indicated that *B. afzelii* does not exhibit host specificity. One might argue, however, that using animal tissues will allow the detection of *Borrelia* genotypes that cause systemic infection in the rodents but will miss an important fraction of the *B. afzelii* genotypes: those transmitted *via* co-feeding [18]. In order to circumvent this potential bias, we tested engorged larvae collected from small mammals. Our analysis indicated that most IGS, *dbpA* and *ospC B. afzelii* haplotypes are maintained by both wood mice and bank voles (Fig. 1). Furthermore, the high degree of linkage disequilibrium between the alleles at the three loci implies that the horizontal gene transfer does not occur often in these bacteria and that the spirochetes have evolved to have equal fitness for both species of the main rodent hosts. This implies that the innate immune response of the various small rodents exempts a more or less similar selective pressure on *B. afzelii*. The absence or scarcity of a haplotype in a rodent species in our study could be the result of a small sample size, so that the sampled rodents were infected with some haplotypes only, due to chance. On the other hand, our sample size could have been insufficient to capture the whole spectrum of haplotypes, so that we cannot exclude the possibility that other haplotypes of *B. afzelii* exist and those would show a different distribution pattern across the vertebrate hosts.

Another reason for the genetic variation at the selected loci could be, as pointed out previously [13, 27], geographical differentiation. Our data indicated that there is genetic variation among the sampling locations but this is lower than the genetic variation within the locations for all three genetic loci (Table 2). We conclude, thus, that geographical differentiation at the local scale of *B. afzelii* strains at these three loci plays a minor role in the observed polymorphism. There are, however, differences in the frequencies of the haplotypes at various sampling sites. This can be explained by a negative frequency-dependent mechanism in which no genotype has a maximum fitness within a certain host species but that initial infection of a host triggers an immune response that will be protective against subsequent infections with genetically similar bacterial genotypes [42, 43]. The genotype that is most abundant at some point in time will be gradually decreased in frequency by negative selection from the host, favouring another one to become more frequent, with the ensuing fluctuations in time and space of the genotype’s frequencies [44].

In general, a third reason for the maintenance of the genetic variation at some genetic loci of *B. afzelii* could be the interaction bacterium-tick species. While some *Ixodes* species transmit multiple *B. burgdorferi* (*s.l.*) genospecies, other tick-*Borrelia* associations seem to be less efficient [45]. Different tick receptors for *Borrelia* proteins could account for different attachment rates of the spirochaetes to the tick midgut and, hence, for their abundance in enzootic cycles [46–49]. Since in our study we only looked at *I. ricinus* larvae, which were only very seldom co-feeding with other *Ixodes* spp., we can exclude the possibility of selection of *Borrelia* genotypes by the tick species.

Several studies have shown that there are *B. afzelii* multilocus sequence types that are overrepresented in human samples, as compared to the tick samples [7, 50]. The natural question that ensues is: What is the provenience of those sequence types? Is there a vertebrate host in nature that can act as main reservoir for these sequence types? Our study suggests that the differential virulence of the *B. afzelii* sequence types is not the result of maintenance of the bacteria in distinct vertebrate hosts in enzootic cycles. The various rodent species were capable of transmitting or allowing transmission of many overlapping *B. afzelii* haplotypes. This finding, together with the variety of the transmission cycles of *B. afzelii* throughout Europe [3], have direct implications for the control of *B. afzelii*, in the sense that interventions on the diversity of small mammal hosts would have limited, if any, effect in reducing the density of ticks infected with a certain bacterial haplotype. This is in contrast to what the “dilution effect”, according to which more diverse host communities would lead to a reduction of the strains with increased pathogenicity for humans due to a decrease in the relative abundances of the competent reservoir hosts [10–12], would predict. Instead, the overall number of ticks feeding on the rodents could be decreased indirectly, by increasing the number of predators [28] or excluding propagation hosts (e.g. deer) at small spatial scales [51].

## Conclusions

We show that small rodents do not contribute differently to the genetic diversity of *B. afzelii* at neither chromosomal nor plasmid loci. The results of the genetic analysis indicate that the existence of multiple haplotypes at plasmid loci that are responsible for the microorganism-host interaction is unlikely to be the result of niche-polymorphism balancing selection. It is possible that another form of balancing selection, such as negative frequency-dependent selection, is responsible for maintaining the genetic diversity at various loci in the *B. afzelii* genome. Further studies are necessary to elucidate the ecological factors that drive and maintain the genetic differentiation of Lyme disease spirochetes at an intraspecific level.

## Additional files

Additional file 1: **Table S1.** Location of sampling sites. (CSV 688 bytes)

Additional file 2: **Table S2.** Tick burdens of rodents and *B. burgdorferi* (*s.l*.) prevalence of infection in tested ticks. (CSV 9 kb)

Additional file 3: **Table S3.** Infestation level of small mammals with *I. ricinus* ticks: centrality and dispersion descriptors. (CSV 757 bytes)

Additional file 4: **Table S4.** Primers and probes used in the qPCR analyses. (CSV 824 bytes)

Additional file 5: **Table S5.** Primers for conventional PCR and sequencing. (CSV 677 bytes)

Additional file 6: **Table S6.** Haplotype sequences of IGS, *dbpA,* and *ospC* found in the ticks sampled from the three rodent species. (CSV 201 kb)

Additional file 7: **Table S7.** Distribution of haplotypes over the ticks sampled from the three rodent species. (CSV 1 kb)

## Abbreviations

*DbpA*: decorin binding protein A
*FlaB*: flagellin B
IGS: 5S-23S rDNA intergenic spacer region
*OspA*: outer surface protein A
*OspC*: outer surface protein C
TBP: tick-borne pathogens

## References

[1] Stanek G, Wormser GP, Gray J, Strle F. Lyme borreliosis. Lancet. 2012;379:461–73. 10.1016/S0140-6736(11)60103-7 21903253

[2] Rauter C, Hartung T (2005). Prevalence of *Borrelia burgdorferi sensu lato* genospecies in *Ixodes ricinus* ticks in Europe: a metaanalysis. Appl Environ Microbiol..

[3] Hofmeester TR, Coipan EC, SEv W, Prins HHT, Takken W, Sprong H (2016). Few vertebrate species dominate the *Borrelia burgdorferi s.l.* life cycle. Environ Res Lett..

[4] Dykhuizen DE, Brisson D, Sandigursky S, Wormser GP, Nowakowski J, Nadelman RB (2008). The propensity of different *Borrelia burgdorferi sensu stricto* genotypes to cause disseminated infections in humans. Am J Trop Med Hyg..

[5] Strle K, Jones KL, Drouin EE, Li X, Steere AC (2011). *Borrelia burgdorferi* RST1 (OspC type A) genotype is associated with greater inflammation and more severe Lyme disease. Am J Pathol..

[6] Wormser GP, Brisson D, Liveris D, Hanincova K, Sandigursky S, Nowakowski J (2008). *Borrelia burgdorferi* genotype predicts the capacity for hematogenous dissemination during early Lyme disease. J Infect Dis..

[7] Coipan EC, Jahfari S, Fonville M, Oei GA, Spanjaard L, Takumi K (2016). Imbalanced presence of *Borrelia burgdorferi s.l.* multilocus sequence types in clinical manifestations of Lyme borreliosis. Infect Genet Evol..

[8] Levene H (1953). Genetic equilibrium when more than one ecological niche is available. Am Nat..

[9] Gliddon C, Strobeck C (1975). Necessary and sufficient conditions for multiple-niche polymorphism in haploids. Am Nat..

[10] States SL, Brinkerhoff RJ, Carpi G, Steeves TK, Folsom-O'Keefe C, DeVeaux M (2014). Lyme disease risk not amplified in a species-poor vertebrate community: similar *Borrelia burgdorferi* tick infection prevalence and OspC genotype frequencies. Infect Gen Evol..

[11] Norman R, Bowers RG, Begon M, Hudson PJ. Persistence of tick-borne virus in the presence of multiple host species: tick reservoirs and parasite-mediated competition. J Theor Biol. 1999;200:111–8. 10.1006/jtbi.1999.0982 10479543

[12] Schmidt KA, Ostfeld RS (2001). Biodiversity and the dilution effect in disease ecology. Ecology..

[13] Hellgren O, Andersson M, Raberg L (2011). The genetic structure of *Borrelia afzelii* varies with geographic but not ecological sampling scale. J Evol Biol..

[14] Jacquot M, Bisseux M, Abrial D, Marsot M, Ferquel E, Chapuis JL (2014). High-throughput sequence typing reveals genetic differentiation and host specialization among populations of the *Borrelia burgdorferi* species complex that infect rodents. PLoS One..

[15] Durand J, Jacquet M, Paillard L, Rais O, Gern L, Voordouw MJ (2015). Cross-immunity and community structure of a multiple-strain pathogen in the tick vector. Appl Environ Microbiol..

[16] Raberg L, Hagstrom A, Andersson M, Bartkova S, Scherman K, Strandh M (2017). Evolution of antigenic diversity in the tick-transmitted bacterium *Borrelia afzelii*: a role for host specialization?. J Evol Biol..

[17] Perez D, Kneubuhler Y, Rais O, Jouda F, Gern L (2011). *Borrelia afzelii* ospC genotype diversity in *Ixodes ricinus* questing ticks and ticks from rodents in two Lyme borreliosis endemic areas: contribution of co-feeding ticks. Ticks Tick Borne Dis..

[18] Tonetti N, Voordouw MJ, Durand J, Monnier S, Gern L (2015). Genetic variation in transmission success of the Lyme borreliosis pathogen *Borrelia afzelii*. Ticks Tick Borne Dis..

[19] Brisson D, Dykhuizen DE (2004). ospC diversity in *Borrelia burgdorferi*: different hosts are different niches. Genetics..

[20] Mechai S, Margos G, Feil EJ, Barairo N, Lindsay LR, Michel P (2016). Evidence for host-genotype associations of *Borrelia burgdorferi sensu stricto*. PLoS One..

[21] Piesman J, Gern L. Lyme borreliosis in Europe and North America. Parasitology. 2004;129(Suppl.):S191–220. 10.1017/s0031182003004694 15938512

[22] Gassner F, Takken W, Plas CL, Kastelein P, Hoetmer AJ, Holdinga M (2013). Rodent species as natural reservoirs of *Borrelia burgdorferi sensu lato* in different habitats of *Ixodes ricinus* in The Netherlands. Ticks Tick Borne Dis..

[23] De Boer R, Hovius KE, Nohlmans MK, Gray JS (1993). The woodmouse (*Apodemus sylvaticus*) as a reservoir of tick-transmitted spirochetes (*Borrelia burgdorferi*) in The Netherlands. Zentralbl Bakteriol..

[24] Hanincova K, Schafer SM, Etti S, Sewell HS, Taragelova V, Ziak D (2003). Association of *Borrelia afzelii* with rodents in Europe. Parasitology..

[25] Wilske B, Preac-Mursic V, Schierz G, Busch KV (1986). Immunochemical and immunological analysis of European *Borrelia burgdorferi* strains. Zentralbl Bakteriol Mikrobiol Hyg A..

[26] Cassatt DR, Patel NK, Ulbrandt ND, Hanson MS (1998). DbpA, but not OspA, is expressed by *Borrelia burgdorferi* during spirochetemia and is a target for protective antibodies. Infect Immun..

[27] Coipan EC, Fonville M, Tijsse-Klasen E, van der Giessen JW, Takken W, Sprong H (2013). Geodemographic analysis of *Borrelia burgdorferi sensu lato* using the 5S-23S rDNA spacer region. Infect Genet Evol..

[28] Hofmeester TR, Jansen PA, Wijnen HJ, Coipan EC, Fonville M, Prins HHT, et al. Cascading effects of predator activity on tick-borne disease risk. Proc Biol Sci. 2017;284:20170453. 10.1098/rspb.2017.0453 PMC5543215 28724731

[29] van Duijvendijk G, Coipan C, Wagemakers A, Fonville M, Ersoz J, Oei A (2016). Larvae of I*xodes ricinus* transmit *Borrelia afzelii* and *B. miyamotoi* to vertebrate hosts. Parasit Vectors..

[30] Silaghi C, Beck R, Oteo JA, Pfeffer M, Sprong H (2016). Neoehrlichiosis: an emerging tick-borne zoonosis caused by “*Candidatus* Neoehrlichia mikurensis”. Exp Appl Acarol..

[31] Qiu WG, Dykhuizen DE, Acosta MS, Luft BJ (2002). Geographic uniformity of the Lyme disease spirochete (*Borrelia burgdorferi*) and its shared history with tick vector (*Ixodes scapularis*) in the Northeastern United States. Genetics..

[32] Sanger F, Nicklen S, Coulson AR (1977). DNA sequencing with chain-terminating inhibitors. Proc Natl Acad Sci USA..

[33] Katoh K, Standley DM (2013). MAFFT multiple sequence alignment software version 7: improvements in performance and usability. Mol Biol Evol..

[34] Martin D, Rybicki E (2000). RDP: detection of recombination amongst aligned sequences. Bioinformatics..

[35] Yu G, Smith DK, Zhu H, Guan Y, TT-Y L (2017). ggtree: an R package for visualization and annotation of phylogenetic trees with their covariates and other associated data. Methods Ecol Evol..

[36] Excoffier L, Lischer HE (2010). Arlequin suite ver 3.5: a new series of programs to perform population genetics analyses under Linux and Windows. Mol Ecol Resour..

[37] R Core Team. A language and environment for statistical computing. Vienna. Austria: R Foundation for Statistical Computing; 2018.

[38] Richter D, Debski A, Hubalek Z, Matuschka FR (2012). Absence of Lyme disease spirochetes in larval *Ixodes ricinus* ticks. Vector Borne Zoonotic Dis..

[39] Rollend L, Fish D, Childs JE (2013). Transovarial transmission of *Borrelia spirochetes* by *Ixodes scapularis*: a summary of the literature and recent observations. Ticks Tick Borne Dis..

[40] Talleklint L, Jaenson TG (1994). Transmission of *Borrelia burgdorferi s.l.* from mammal reservoirs to the primary vector of Lyme borreliosis, *Ixodes ricinus* (Acari: Ixodidae), in Sweden. J Med Entomol..

[41] Humair PF, Rais O, Gern L (1999). Transmission of *Borrelia afzelii* from *Apodemus* mice and *Clethrionomys* voles to *Ixodes ricinus* ticks: differential transmission pattern and overwintering maintenance. Parasitology..

[42] Gromko MH (1977). What is frequency-dependent selection?. Evolution..

[43] Barthold SW (1999). Specificity of infection-induced immunity among *Borrelia burgdorferi sensu lato* species. Infect Immun..

[44] Kurtenbach K, Hanincova K, Tsao JI, Margos G, Fish D, Ogden NH (2006). Fundamental processes in the evolutionary ecology of Lyme borreliosis. Nat Rev Microbiol..

[45] Masuzawa T, Kharitonenkov IG, Kadosaka T, Hashimoto N, Kudeken M, Takada N (2005). Characterization of *Borrelia burgdorferi sensu lato* isolated in Moscow province – a sympatric region for *Ixodes ricinus* and *Ixodes persulcatus*. Int J Med Microbiol..

[46] Konnai S, Yamada S, Imamura S, Nishikado H, Githaka N, Ito T (2012). Identification of TROSPA homologue in *Ixodes persulcatus* Schulze, the specific vector for human Lyme borreliosis in Japan. Ticks Tick Borne Dis..

[47] Figlerowicz M, Urbanowicz A, Lewandowski D, Jodynis-Liebert J, Sadowski C (2013). Functional insights into recombinant TROSPA protein from *Ixodes ricinus*. PLoS One..

[48] Coipan C, Sprong H. Ecology of *Borrelia burgdorferi sensu lato*. In: MAH B, van Wieren SE, Takken W, Sprong H, editors. Ecology and Control of Vector-borne Diseases, Vol. 4. Wageningen, The Netherlands: Wageningen Academic Publishers; 2016. p. 41–61.

[49] Estrada-Pena A, Sprong H, Cabezas-Cruz A, de la Fuente J, Ramo A, Coipan EC (2016). Nested coevolutionary networks shape the ecological relationships of ticks, hosts, and the Lyme disease bacteria of the *Borrelia burgdorferi* (*s.l.*) complex. Parasit Vectors..

[50] Jungnick S, Margos G, Rieger M, Dzaferovic E, Bent SJ, Overzier E (2015). *Borrelia burgdorferi sensu stricto* and *Borrelia afzelii*: Population structure and differential pathogenicity. Int J Med Microbiol..

[51] Hofmeester TR, Sprong H, Jansen PA, Prins HHT, van Wieren SE (2017). Deer presence rather than abundance determines the population density of the sheep tick, *Ixodes ricinus*, in Dutch forests. Parasit Vectors..

